# RARRES3 expression positively correlated to tumour differentiation in tissues of colorectal adenocarcinoma

**DOI:** 10.1038/sj.bjc.6601049

**Published:** 2003-07-01

**Authors:** R-Y Shyu, S-Y Jiang, J-M Chou, Y-L Shih, M-S Lee, J-C Yu, P-C Chao, Y-J Hsu, S-W Jao

**Affiliations:** 1 Department of Internal Medicine, Tri-Service General Hospital, 325 Chengung Rd, Sec. 2, Taipei, Taiwan 114, Republic of China; 2 Department of Microbiology and Immunology, National Defense Medical Center, 161 Minchuan East Rd, Sec. 6, Taipei, Taiwan 114, Republic of China; 3 Department of Pathology, Tri-Service General Hospital, 325 Chengung Rd, Sec. 2, Taipei, Taiwan 114, Republic of China; 4 School of Public Health, National Defense Medical Center, 161 Minchuan East Rd, Sec. 6, Taipei, Taiwan 114, Republic of China; 5 Department of Surgery, Tri-Service General Hospital, 325 Chengung Rd, Sec. 2, Taipei, Taiwan 114, Republic of China

**Keywords:** RARRES3, RIG1, TIG3, colorectal carcinoma, differentiation, tumour suppressor gene

## Abstract

*RARRES3* is a retinoid-inducible class II tumour-suppressor gene. This study analysed the expression of RARRES3 protein in normal, adenoma and carcinoma tissues of the colorectum and its correlation with tumour differentiation. The expression of RARRES3 protein in 151 paraffin-embedded colorectal tissues (11 distal normal mucosa, 20 adenoma and 120 colorectal adenocarcinoma) was determined by immunohistochemistry. RARRES3 protein was expressed in all 11 distal normal, 120 adjacent normal and 20 adenoma tissues. In distal normal tissues, RARRES3 protein was expressed at the highest levels in differentiated mucosal epithelial cells. Among 120 carcinoma tissues, RARRES3 protein was detected in 97.6% (40 out of 41), 79.4% (54 out of 68) and 17.3% (three out of 11) of well-, moderately and poorly differentiated tumours, respectively. The expression of RARRES3 protein was positively correlated to tumour differentiation (test for trend, *P*<0.0001). Also, levels of RARRES3 protein were found to be higher in the normal tissues adjacent to 14.6% (six out of 41), 51.5% (35 out of 68), and 90.1% (10 out of 11) of well-, moderately and poorly differentiated tumours, respectively. The decreases in tumour differentiation and RARRES3 expression were significantly correlated compared to the adjacent normal tissues (test for trend, *P*<0.0001). The prognostic implication of RARRES3 protein expression was studied in 107 tumour, and no statistical difference in survival was observed. The expression of RARRES3 protein was positively correlated to cellular differentiation of normal and adenocarcinoma tissues of the colorectum, which supports the role of RARRES3 in normal and malignant epithelial differentiation of colorectum. RARRES3 expression was decreased only in carcinoma tissue, which suggests that altered RARRES3 expression occurs late in colorectal carcinogenesis.

Retinoid acid receptor responder 3 (*RARRES3*), also named as *TIG3* (DiSepio *et al*, 1998) or *RIG1* (Huang *et al*, 2000), was isolated from retinoid-treated cells using mRNA differential display. cDNA of *RARRES3* encodes an 18-kDa protein with 164 amino acids. *RARRES3* along with *HREV107* (Hajnal *et al*, 1994; Husmann *et al*, 1998)and *A-C1* (Akiyama *et al*, 1999) belong to a family of class II tumour-suppressor genes that block reversible expression rather than sustained mutation, as a general mechanism of gene inactivation (Sager R, 1997). Proteins of the *HREV107* family are shown to suppress transformation induced by H-*ras* (Hajnal *et al*, 1994; Sers *et al*, 1997; Akiyama *et al*, 1999) or kinase activities downstream of the activation of Ras proteins (Huang *et al*, 2002). The *RARRES3* gene is expressed ubiquitously in normal tissues, and cancer cell lines have low *RARRES3* expression. Ectopic expression of RARRES3 in cancer cells leads to growth suppression or cellular apoptosis (DiSepio *et al*, 1998; Deucher *et al*, 2000; Huang *et al*, 2002).

Differential expression of RARRES3 in benign and malignant tissues has not been extensively studied. RARRES3 expression is reduced in tissues of basal cell carcinoma and aggressive squamous cell carcinoma (Duvic *et al*, 2000). Also, the expression of RARRES3 is downregulated in B-cell lymphocytic leukaemias with disease progression (Casanova *et al*, 2001). Similarly, the expression of HREV107 is positively correlated to cellular differentiation. The rat HREV107 protein is expressed in differentiated epithelial cells of the gastrointestinal tract, and many cancer cell lines have downregulated HREV107 expression (Sers *et al*, 1997). HREV107 is expressed in postmeiotic human testicular germ cells (Siegrist *et al*, 2001). Therefore, RARRES3 and HREV107 may be important in epithelial differentiation, and altered expression of these proteins may play an important role in carcinogenesis.

Mechanisms of colorectal tumorigenesis have been studied extensively. Mutations in the adenomatous polyposis coli tumour-suppressor gene were proposed to occur early during the development of polyps, and oncogenic *KRAS* mutations arose during the adenomatous stage. The presence of mutations of *TP53*, deletions on chromosome 18q (where tumour-suppressor genes *DCC*, *SMAD2* and *SMAD4* have been localised) and mutations of DNA mismatch repair genes coincided with the transition to malignancy (Vogelstein *et al*, 1988; Chung, 2000). The *RARRES3* gene is localised at chromosome 11q12 (Auer *et al*, 2002), where deletion has been observed in tissues of lung and ovary cancer (Gabra *et al*, 1995; Iizuka *et al*, 1995). To investigate the expression and role of RARRES3 protein in colorectal carcinogenesis, we have analysed the expression of RARRES3 protein in tissues from the normal colorectal mucosa, and from adenomas and adenocarcinomas using immunohistochemistry (IHC).

## MATERIALS AND METHODS

### Preparation of RARRES3 antiserum

The RARRES3 peptide corresponding to amino acids 74–87 of the RARRES3 protein (Huang *et al*, 2000) was synthesised and conjugated to keyhole limpet haemocyanin (Genosys Biotechnologies Inc, Woodlands, TX, USA). In total, 200 *μ*g of conjugated peptide antigen mixed thoroughly with Freund's complete adjuvant was injected into the popliteal lymph node of New Zealand white rabbits. The injection was repeated every 2 weeks (a total of six injections) and consisted of 100-*μ*g RARRES3 peptide mixed with the incomplete Freund's adjuvant. Titres of the antiserum were determined using an enzyme immunoassay. The specificity of the antiserum was determined by Western blotting of cytosol extracts prepared from cells expressing the RARRES3-fusion protein (Huang *et al*, 2002).

### Specimen collection and preparation

A total of 20 adenoma tissues were obtained from 14 male and six female patients with mean age of 63.3 years. A total of 120 primary adenocarcinoma with adjacent normal tissues (four from the caecum, 115 from the colon and one from the rectum) were obtained from 68 male and 52 female patients with a mean age of 64.2 years. The distribution of tumours according to the level of differentiation and Dukes' stages are listed in Table 1

**Table 1:** Expression of RARRES3 protein in colorectal tissues

		**RARRES3 protein levels (No. (%) of cases)**			**IRS^a^**
**Tissues**	**No. of cases**	**Negative**	**Weak**	**Strong**	**Mean±s.e.m.**
Distal normal	11	0	3 (27.3)	8 (72.7)	5.55±1.76
Adenoma	20				7.80±1.51
Mild dysplasia	11	0	0	11 (100)	
Moderate dysplasia	8	0	0	8 (100)	
Severe dysplasia	1	0	0	1 (100)	
Tumours					
Adjacent normal	120	0	7 (5.8)	113 (94.2)	7.16±1.85
Adenocarcinoma	120				
Differentiation					
Well	41	1 (2.4)	5 (12.2)	35 (85.4)	7.05±2.36^a^
Moderate	68	14 (20.6)	23 (33.8)	31 (45.6)	4.68±3.20^b^
Poor	11	8 (72.7)	2 (18.2)	1 (9.1)	1.36±1.96^c^
Duke's stages					
A+B	51	8 (15.7)	12 (23.5)	31 (60.8)	5.71±3.30
C	44	8 (18.2)	11 (25.0)	25 (56.8)	5.23±3.16
D	25	7 (28.0)	7 (28.0)	11 (44.0)	4.04±3.19

a IRS=immunoreactive score.

The values of IRS with different superscripts are significantly different using Kruskal–Wallis test followed by Dunn's procedure, *P*<0.05.

. In addition, 11 distal normal tissues were taken from regions >10 cm away from the bulk of those tumour tissues that had clearly defined margins. Tissue slides were prepared from paraffin-embedded blocks with haematoxylin and eosin staining. Each specimen was evaluated by the same pathologist to define the differentiation status of carcinoma tissues as well as the degree of dysplasia of adenoma tissues. The assessment of tumour differentiation is based on the architectural and glandular differentiation as well as nuclear features of tumours (Anonymous, 1996). Primary tumours were staged according to Dukes' classification system (Dukes, 1932).

### Immunohistochemical analysis

Tissue sections were air-dried, deparaffinised, and then boiled twice for 2 min in 10% DAKO ChemMate™ solution (DAKO Co, Carpinteria, CA, USA) containing 0.05% NP-40 (Nonidet P-40). The DAKO LSAB®2 Peroxidase kit was used to stain the expression of RARRES3 protein in tissue sections. Tissues were incubated with RARRES3 antiserum or preimmune serum at a dilution of 1 to 800 at room temperature for 1 h. The sections were lightly counterstained with Mayer's haematoxylin. To verify antibody specificity, RARRES3 antisera were preincubated with 8–30 *μ*g of RARRES3 peptide at 4°C overnight. Samples were spun at 100 000 *g* at 4°C for 10 min before adding the absorbed antisera to the tissue section. Sections were also stained with p21^WAF1^ and Ki-67 monoclonal antibodies obtained from DAKO Co.

### Reviewing and scoring the sections

Patterns of staining, cellular RARRES3 localisation, staining intensity and percentage of RARRES3 expressed cells were recorded. The evaluation of staining patterns was performed using the immunoreactive score (IRS) proposed by Remmele and Stegner (1987), in which IRS = SI (staining intensity) × PP (percentage of positive cells). SI was determined as 0, negative; 1, weak; 2, moderate; and 3, strong. PP was defined as 1, 0–9% positive cells; 2, 10–50% positive cells; and 3, >50% positive cells. A total of 10 high-power visual fields, with 100 cells per field counted from different area a of each specimen were chosen at random for IRS evaluation, and the average of the IRS was calculated. The final intensity of RARRES3 staining was defined as ‘negative,’ ‘weak’ and ‘strong,’ corresponding to IRS values of 0–1, 2–4 and 6–9, respectively (Saukkonen *et al*, 2001). Results of RARRES3 staining were based on the consensus of the two investigators (S-Y J, J-M C). Differences in RARRES3 expression between adjacent normal and carcinoma tissues were based on final RARRES3 staining.

### Statistical analysis

The nonparametric Kruskal–Wallis tests were applied to compare IRS of RARRES3 associated with tumours at various levels of differentiation as well as Dukes' stages. Further, Dunn's procedure was applied to compare the IRS between groups. Logistic regression analyses were used to assess the association and trend between tumour differentiation and chance of positive RARRES3 staining and chance of adjacent normal tissues having RARRES3 expression higher than that of tumours, while controlling for potential confounding factors, that is, subject's gender and age. Survival rates were calculated using the Kaplan–Meier method. Significance was calculated by the log-rank test. To further validate the effect of RARRES3 staining on survival, a multivariate Cox proportional hazard method was used to adjust for age and stage.

## RESULTS

### Analysis of RARRES3 antisera

**Figure 1:**
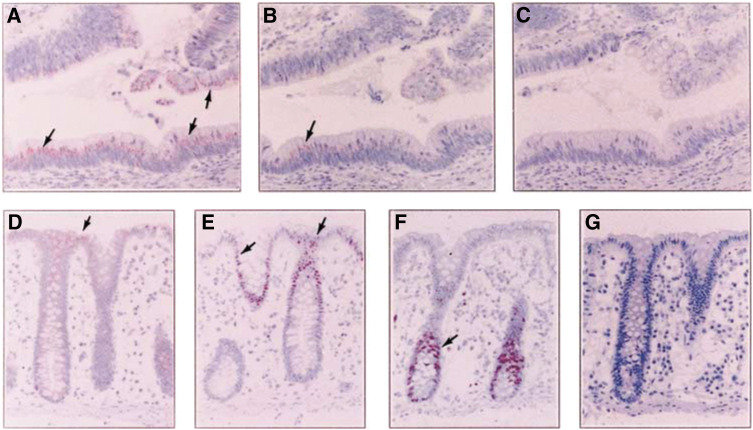
Immunohistochemical analysis of the specificity of RARRES3 antiserum and expression of RARRES3, p21^WAF1^ and Ki-67 proteins in colon mucosal tissues. Sections of well-differentiated adenocarcinoma of colon were incubated with RARRES3 antiserum (**A**), RARRES3 antiserum preincubated with 10 *μ*g of RARRES3 peptide (**B**) or preimmune serum (**C**). Magnification X200. Sections from distal normal mucosal tissues of colon were incubated with antibodies against RARRES3 (**D**), p21^WAF1^ (**E**), Ki-67 (**F**) or preimmune serum (**G**). Magnification X100. Sections were counterstained with Mayer's haematoxylin. Arrows indicate positive staining of RARRES3, p21^WAF1^ or Ki-67.

The specificity of RARRES3 antiserum was tested on well-differentiated colon adenocarcinoma tissue using IHC. Tissues incubated with RARRES3 antiserum showed RARRES3-positive and granular staining localised at the supranuclear regions of tumour, adenoma and normal mucosal cells (Figures 1

and 2

**Figure 2:**
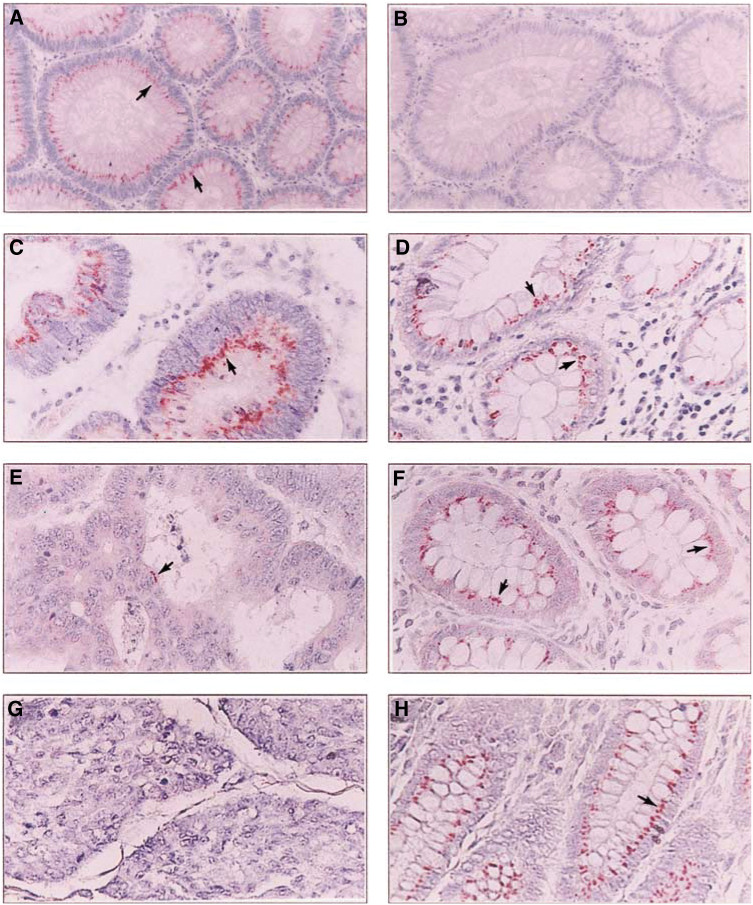
Expression of RARRES3 protein in adenoma, carcinoma and adjacent normal tissues. Paraffin-embedded sections of adenoma (**A**, **B**), well- (**C**, **D**), moderately (E, F) and poorly (**G**, **H**) differentiated colorectal carcinoma were assayed for RARRES3 protein expression by IHC using RARRES3 antiserum (**A**, **C**–**H**) or preimmune serum (**B**). Expression of RARRES3 protein in adenoma (**A**), carcinoma (**C**, **E**, and **G**) and adjacent normal tissues (**D**, **F**, **H**) are shown. Sections were counterstained with Mayer's haematoxylin. Original magnification X100 (**A**, **B**) and X200 (**C–H**). Arrows indicate positive RARRES3 staining.

). Some random granular patterns of RARRES3 staining were observed in tumour cells. The staining was specific, since preincubation of the antiserum with 10 *μ*g of RARRES3 peptide resulted in the suppression of staining (Figure 1B). No specific RARRES3 staining was observed in tissues incubated with preimmune serum (Figure 1C).

### RARRES3 expression in normal and adenoma tissues

A total of 11 distal normal tissues and 120 adjacent normal tissues of carcinomas were analysed for RARRES3 expression by IHC (Table 1). A total of 72.7 and 94.2% of distal normal and normal tissues adjacent to carcinomas, respectively, had strong RARRES3 expression with a mean IRS of 5.55 and 7.16, respectively. The expression of RARRES3, the cell cycle inhibitor p21^WAF1^ and nuclear protein Ki-67 was also analysed in 11 distal normal tissues. The RARRES3 protein was expressed at the highest levels in terminal-differentiated mucosal epithelial cells, which expressed the p21^WAF1^ protein (Figures 1D, E). The less-differentiated and proliferative mucosal crypt cells that were stained positive for the nuclear protein Ki-67 had decreased or exhibited no RARRES3 expression (Figures 1D, F). RARRES3 expression in 20 adenoma tissues with dysplasias ranging from mild to severe was analysed. All 20 adenoma tissues had strong RARRES3 expression regardless of the variation in the degree of dysplasia (Table 1, Figure 2A).

### RARRES3 expression in tissues of colorectal adenocarcinoma

Levels of RARRES3 protein varied among 120 tissues of colorectal adenocarcinoma (Table 1). When RARRES3 expression was analysed with respect to difference in tumour differentiation, 35 out of 41 (85.4%) well-differentiated tissues had strong RARRES3 expression. Among 68 moderately differentiated tumor tissues, 14 tumours (20.6%) did not express RARRES3 protein and 31 tumours (45.6%) had strong RARRES3 expression. Furthermore, eight out of 11 (72.7%) poorly differentiated colorectal carcinoma tissues did not express the RARRES3 protein, and two tissues had weak RARRES3 expression. Representative results of RARRES3 protein expression in well-, moderately and poorly differentiated tumour tissues are shown in Figure 2. RARRES3 protein levels in terms of IRS of various differentiations were significantly different (*P*<0.0001). The better the tumour tissue differentiation, the higher the IRS was rated. A significant linear trend was found in tumour differentiation and RARRES3 expression (*P*<0.0001). No significant association was found between RARRES3 IRS and Dukes' stages.

We also compared levels of RARRES3 protein between adjacent normal and tumour tissues within the same tissue slide among 120 carcinoma tissues (Table 2

**Table 2:** Comparison of RARRES3 protein expression between adjacent normal and adenocarcinoma tissues

		**No. of cases (% of total)**		
**Differentiation**	**No. of cases**	**N>T^a^**	**N=T^b^**	**M<T^c^**
Well	41	6 (14.6)	33 (80.5)	2 (4.9)
Moderate	68	35 (51.5)	31 (45.6)	2 (2.9)
Poor	11	10 (90.1)	1 (9.1)	0 (0)
				
Total cases	120	51 (42.9)	65 (54.2)	4 (3.3)

a Final RARRES3 staining in normal tissues was higher than that of tumour tissues.

b Final RARRES3 staining in normal tissues was similar to that of tumour tissues.

c Final RARRES3 staining in normal tissues was lower than that of tumour tissues.

, Figure 2C–H). Levels of RARRES3 expression in 33 out of 41 (80.5%) well-differentiated tissues were similar to that of the adjacent normal tissues, and six tissues (14.6%) had RARRES3 expression in normal tissues greater than that of tumour tissues. The percentage of tissues that showed higher levels of RARRES3 expression in adjacent normal tissues was increased to 51 and 90.1% in moderately and poorly differentiated adenocarcinoma tissues, respectively. Compared to well-differentiated tumours, moderately and poorly differentiated carcinoma tissues had a significantly increased chance of having higher RARRES3 protein levels in the adjacent normal tissues than in tumour tissues (*P* for trend <0.0001).

### Prognostic impact of RARRES protein expression

**Figure 3:**
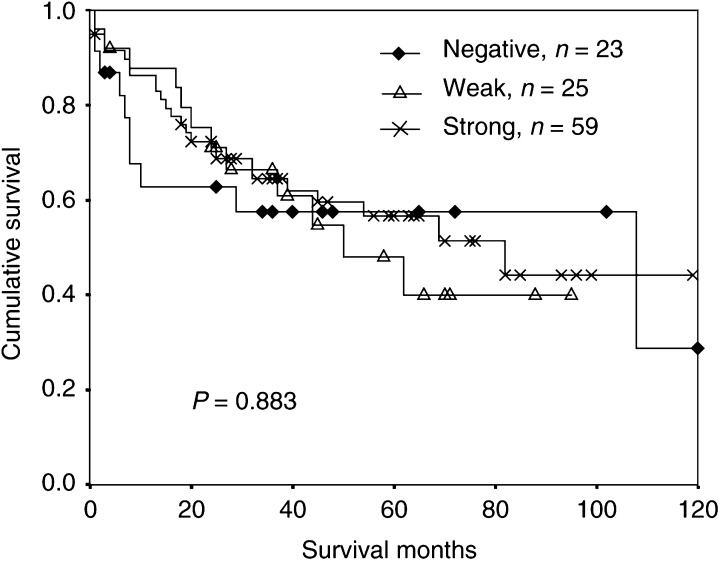
Intensity of RARRES3 staining and overall survival in colorectal cancer patients calculated by the Kaplan–Meier method.

Altogether, 107 patients with colorectal adenocarcinoma were examined for prognosis related to RARRES3 expression. A total of 23 tumours were stained negative for RARRES3 protein, 25 tumours had weak RARRES3 expression and 59 tumours had strong RARRES3 expression. Kaplan–Meier survival curves for the different groups are presented in Figure 3

. Based on the 107 patients, no difference in survival was found comparing patients with negative, weak and strong RARRES3 staining in tumours (*P*=0.883). Similarly, multivariate analysis showed no difference in survival between patients with negative and weak (*P*=0.929) or between negative and strong (*P*=0.292) RARRES3 staining in tumours.

## DISCUSSION

RARRES3 is a retinoid-inducible tumour suppressor. This study shows that RARRES3 protein is expressed in mucosal tissues of normal colon and rectum, which is correlated to epithelial differentiation. The premalignant adenoma tissues expressed the RARRES3 protein at high levels. Within tumour tissues, the expression of RARRES3 protein is positively correlated to tumour differentiation. Compared to the adjacent normal mucosal tissues, tumours with moderately and poorly differentiated adenocarcinoma had significantly reduced RARRES3 expression. However, survival analysis showed that RARRES3 protein was not a prognostic marker for patients with colorectal adenocarcinoma.

In tissues of normal colorectal mucosa, RARRES3 protein is expressed at the highest levels in terminal-differentiated epithelial cells, which coincides with a high level of expression of the cell cycle inhibitor p21^WAF1^ (Reed, 1997). The proliferative, Ki-67-positive mucosal crypt cells (Gerdes *et al*, 1984) show low or no RARRES3 expression. Therefore, RARRES3 is correlated to differentiation and growth arrest of normal mucosal epithelial cells of the colorectum. Similarly, the highest RARRES3 expression is found in normal suprabasal epidermis, hair follicles and sebaceous gland. Therefore, RARRES3 may be associated with terminal keratinocyte differentiation and growth arrest occurring in the suprabasal layers (Duvic *et al*, 2000). Similar to results of normal colonic mucosa, the expression of RARRES3 protein is positively correlated to differentiation of adenocarcinoma of the colorectum. Moderately and poorly differentiated colorectal tumours had progressive loss of the RARRES3 expression. In addition, *in vitro* studies investigating the transient expression of RARRES3 protein or fusion proteins also detected the growth suppressive and proapoptotic activity of these proteins in several cancer cells (DiSepio *et al*, 1998; Deucher *et al*, 2000; Huang *et al*, 2002). Therefore, RARRES3 functions as a negative growth regulator. In addition, it may also be important for normal epithelial differentiation. Similar results were also observed for the HREV107 protein, the expression of which is limited to differentiated epithelial cells and the postmeiotic testicular germ cells (Sers *et al*, 1997; Siegrist *et al*, 2001). Currently, a result that shows the active role of RARRES3 or HREV107 in cellular differentiation has not been reported. Our preliminary results demonstrated that SC-M1 gastric cancer cells became enlarged and flatten, similar to retinoic acid-induced differentiation of SC-M1 cells (Shyu *et al*, 1995), after expressing the RARRES3-EGFP fusion protein for 28 days (data not shown). Further investigation will be required to dissect the active or passive role of RARRES3 in epithelial differentiation.

Genetic alterations have been studied extensively during steps of colorectal carcinogenesis. Mutations in the adenomatous polyposis coli and KRAS oncogenes arose in precancerous lesions. Aberrations in TP53, DCC and SMADs coincide with transition to malignancy (Vogelstein *et al*, 1988; Chung *et al*, 2000). In this study, RARRES3 protein was expressed at high levels in tissues of normal colorectal mucosa and the precancerous lesion adenoma. The decrease in RARRES3 expression was only observed in carcinoma tissues, which are unclassified by the Dukes' system, and also observed in tissues at Dukes' stages A and B. Therefore, the decrease or loss of RARRES3 expression may occur in the early stages of colorectal carcinoma. To our knowledge, deletion at chromosome 11q12, where RARRES3 is localised, has not been reported in tissues of colorectal carcinoma. Also, downregulation instead of mutation of the RARRES3 gene was found during progression of B-cell lymphocytic leukaemia (Casanova *et al*, 2001). Similarly, we did not detect any mutation in cDNA of RARRES3 from 11 cancer cell lines (data not shown). Therefore, epigenetic alteration may play an important role in the decrease in RARRES3 expression in colorectal carcinoma tissues. Mutation at KRAS is frequently observed in adenoma tissues (Vogelstein *et al*, 1988). Our previous studies showed that RARRES3 negatively regulated signal pathways of extracellular signal-regulated kinase, c-Jun N-terminal kinase and p38 mitogen-activated kinase, downstream kinases following activation of the Ras protein (Huang *et al*, 2002). It therefore is likely that high levels of RARRES3 expression in adenoma tissues as observed in this study may prevent malignant transformation in cells harbouring mutations of the Ras family genes through negative regulation of downstream signal pathways of Ras. Further studies on the molecular mechanism of RARRES3 will be necessary to resolve the issue.

In conclusion, this and previous studies have demonstrated the close association of RARRES3 expression in differentiated epidermis and colorectal epithelium. Progressive loss of RARRES3 expression in tissues of moderately and poorly differentiated colorectal adenocarcinoma supports the tumour-suppressive role of RARRES3 in moderately and poorly differentiated colorectal cancer. However, RARRES3 alone was not a prognostic marker for colorectal adenocarcinoma. RARRES3 protein is shown to suppress the downstream signal pathway of Ras. The significance of the loss of RARRES3 expression along with mutations in Ras will be investigated in the future.
